# The relationship between the body and the environment in the virtual world: The interpupillary distance affects the body size perception

**DOI:** 10.1371/journal.pone.0232290

**Published:** 2020-04-24

**Authors:** Daisuke Mine, Nami Ogawa, Takuji Narumi, Kazuhiko Yokosawa

**Affiliations:** 1 Department of Psychology, The University of Tokyo, Tokyo, Japan; 2 Graduate School of Engineering, The University of Tokyo, Tokyo, Japan; 3 Graduate School of Information Science and Technology, The University of Tokyo, Tokyo, Japan; 4 JST PRESTO, Tokyo, Japan; University of Milan, ITALY

## Abstract

Previous research suggests that the size of one’s body is used as a metric to scale the external world. On the other hand, the influence of information from the external world on the perception of body size is unclear. It has been suggested that increased inter-pupillary distance (IPD) leads people to perceive the external world as smaller than it actually is. The present study investigated the effect of the IPD on body size perception, and the relationship between the perceived scale of the body and the external world when the IPD is manipulated. To this end, in a virtual environment, we manipulated the IPD as well as the size and presence of participants’ hands, while participant’s eye height was increased vertically. Results showed that, when participants’ eye height was increased and their hands were enlarged, people with a fixed IPD perceived the size of their body to be large (like a giant) while the external world was perceived to be changed minimally. Alternatively, people with increased IPD perceived that the external world as having shrank, whereas their perception of their body size changed little. However, when a viewers’ virtual hands were not shown, the IPD did not affect the individual’s percept of body size, although the IPD did affect one’s perception of the external world. These results suggest that, when the ratio of the size between one’s body and the external world are explicit, the perceived size of one’s body is affected by the IPD or perceived scale of the external world that is affected by the IPD.

## Introduction

In virtual environments, people can perceive a body that does not look like their real body as their own body. This phenomenon is termed the *body ownership illusion* [1, 2]. Hence people can be embodied in a virtual body that is larger (or smaller) than their real body. This feature of virtual environments raises questions concerning how people feel when they become ‘giants’. Do they really feel that they are getting larger? This is a very complex issue. This is because people may interpret such phenomena in two ways: (1) they become giants in the normal sized world, or (2) they remain a normal sized human, but in a miniature world. In these two interpretations, the images presented to their eyes are physically the same. That is, people are seeing the same images, yet interpreting them in a vastly different manner. Therefore, questions about this phenomenon must be addressed at the cognitive level. In Jonathan Swift’s *Gulliver’s Travels* (1726), when Gulliver met the Lilliputians, he perceived that the Lilliputians and their artifacts were very small rather than perceiving himself as a giant. On the other hand, the Lilliputians perceived Gulliver as a giant rather than perceiving themselves as dwarfs. In both cases, these characters believed that their own bodies were natural and normal and used their bodies as metrics to scale the external world.

Much research supports the notion that the size of one’s body is used as a metric to scale the external world, this is known as body-based scaling. van der Hoort, Guterstam, & Ehrsson (2011) showed that people embodied in a large virtual body perceived objects to be smaller and nearer, and conversely, people embodied in a small virtual body perceived objects to be larger and further away [3]. These effects have been observed not only with whole bodies but also with parts of the body, such as a hand. Linkenauger et al. (2013) showed that people perceived objects to be smaller when a virtual hand is large, and vice versa [4]. van der Hoort, & Ehrsson (2014) suggested that, by changing body size, the entire surrounding spatial layout rescales, and this effect is distinct from the role of body as a direct familiar-size cue [5]. Overall, this research suggests that our body is used as a metric to scale the external world.

However, Ogawa, Narumi & Hirose (2017) showed that not only does the size of one’s hands influence the perceived size of the environment, but the size of familiar-sized objects also influences the perception of hand size [6]. These authors suggested the possibility that there is also an effect of the scale of the external world on one’s body size perception. Langbehn et al. (2016) also proposed that the presence of the avatar, the type of the virtual environment (i.e., urban vs. artificial) and the presence of other avatars influence a user’s spatial judgement of the dominant scale (i.e., the metric used to scale the other, namely the body or the environment) [7]. These authors suggested that the environment can affect one’s perception of body size, although interpersonal differences of the effect of these factors on spatial judgement of dominant scale was robustly observed. These studies raised the possibility that the external world affords a metric to scale one’s body. Accordingly, the aim of the present study is to further investigate the relationship between one’s body and the external world.

As described above, perceived body size and the perceived scale of the external world are interdependent as well as indefinite. However, the scale of the external world is affected by many factors other than one’s body size. Much prior research suggests that our perception of the external world is affected by a viewer’s inter-pupillary distance (IPD). So far, the influence of changing the IPD on a viewer’s perception of the size and distance of objects has been investigated by using the telestereoscope or virtual reality. They show wider IPD results in viewer’s underestimation of both object sizes [8–10] and depth distance [8, 11, 12]. These findings suggest that increased IPD makes people perceive the external world to be smaller. However, the effect of the IPD on body size perception has not been examined. Because perceived body size and the perceived scale of the external world are interdependent, if the IPD robustly affects the perception of the external world, it is also possible that the IPD will affect the perception of the perceiver’s own body. Accordingly, the present study is designed to investigate the effect of the IPD on body size perception as well as the relationship between the perceived scale of one’s own body and the external world when the IPD is manipulated. Of most import is that fact that we focused on the effect of the IPD under conditions in which the ratio of the size between the body and the external world is identical. Previous studies on body-based scaling have manipulated only the size of the observer’s body; in other words, they varied the ratio of the size between the body and the external world. Therefore, in line with body-based scaling this suggests if one’s body serves as the metric to scale the external world, then there should be no difference between the perception of the body and the external world regardless of the manipulations of the IPD. However, if alternatively the IPD affects the perception of both the body and the external world, then this perception cannot be explained by body-based scaling.

The first aim of the present study is to investigate the effect of the IPD on size perception of one’s own body. Here, we focus on the perceived size of one’s own body and the perceived scale of the external world separately in order to investigate the relationship between them. Most prior research has focused on the perceived scale of the external world when participants were embodied in a large (or small) virtual body; however, precisely how participants perceived the size of their bodies was not sufficiently investigated. Accordingly, in Experiment 1, we investigated the perception of the human body and that of the external world when the ratio of sizes between these two properties change. Specifically, we assessed the effect of the IPD on these percepts in these situations. Thus, we manipulated participants’ hand size (static or getting larger) and the IPD (static or getting wider) while vertically increasing the participants’ eye height. Participants had to report both the perceived height of their virtual body as well as the perceived scale of the external world. In this scenario, the difference between our research and previous studies is that we focus on both the size of the viewer’s body and the scale of the external world in order to ascertain how the IPD affects the perception of both of them, but especially that of body size perception. Finally, to further investigate interaction effect between presence of body parts and the IPD on the scale perception of body and the external world, Experiment 2 used a factorial 2 × 2 design (presence of hands × IPD).

## General method

### Ethical statement

All participants wrote informed consent before participating the experiments. All experiments in the study were approved by the ethical committee of Department of Psychology, the University of Tokyo. All of the other procedures described below were approved by the same ethical committee, and conducted according to the principles and guidelines of the Declaration of Helsinki.

### Participants

We recruited a total of 59 healthy participants for the two experiments: Experiment 1, 35 participants (10 females, 25 males, ages 19–25 [mean = 21.9 years]); Experiment 2, 24 participants (5 females, 19 males, ages 18–24 [mean = 21.8 years]). All participants had normal or corrected-to-normal stereo vision. We recruited naïve participants for each individual experiment to prevent participants from being influenced by previous experimental experiences.

### Materials

The position and movement of participants’ hands were tracked using a motion tracker (Leap Motion Controller by Ultraleap, ltd; Hand tracking running at 150 fps). Participants saw virtual hands from first person perspective through a head mounted display (HMD: Oculus Rift CV1 which displayed a stereoscopic image with a resolution of 2160×1200), and no other body parts were presented. A virtual world was developed using Unity3D and run on a Windows PC (Level Infinity by iiyama: Intel core i7-7700HQ at 2.8 GHz, 16 GB RAM, and NVIDIA GeForce GTX 1060). The visual stimulus was an outdoor scene based on a Japanese city model in which there are plenty of familiar objects (e.g., buildings, cars and traffic signals). The visual stimulus was displayed at 90 fps.

### Manipulations of the eye height, the IPD and the hand size

In all conditions, we manipulated the eye height from the ground. Before manipulation, the eye height was set to 1.57m, the average standing eye height. Next, the eye height was continuously increased up to 18.84m with constant velocity of 2.5 meter per second. In certain conditions within these experiments, the IPD was manipulated by the following procedure. Before an experiment, each participant adjusted the distance between the lenses, used in the HMD, to fit the distance between the pupils of him/her. Before the manipulation of the IPD, the distance between the cameras that render the stereo viewpoints was set to 6.43 cm. Then, the distance between rendering cameras was continuously increased up to 77.16cm corresponding to the continuous manipulation of the eye height. In some conditions, the size of the virtual hands was also manipulated. Before manipulation, the size of the virtual hands was set to the size of each participant’s real hands. Then, the hand size was continuously increased to be twelve times greater than initial hand size corresponding to the manipulation of the eye height. Therefore, the magnification of these components was equal, e.g., when the eye height was 3.14 m (i.e., two times initial eye height), the IPD was 12.86 cm (i.e., two times initial IPD) and the size of the virtual hands was two times initial hand size. Similar procedures have been applied in previous research in which the eye height and the IPD were continuously increased (see [10]).

## Experiment.1

### Method

#### Procedure

**Fig 1:**
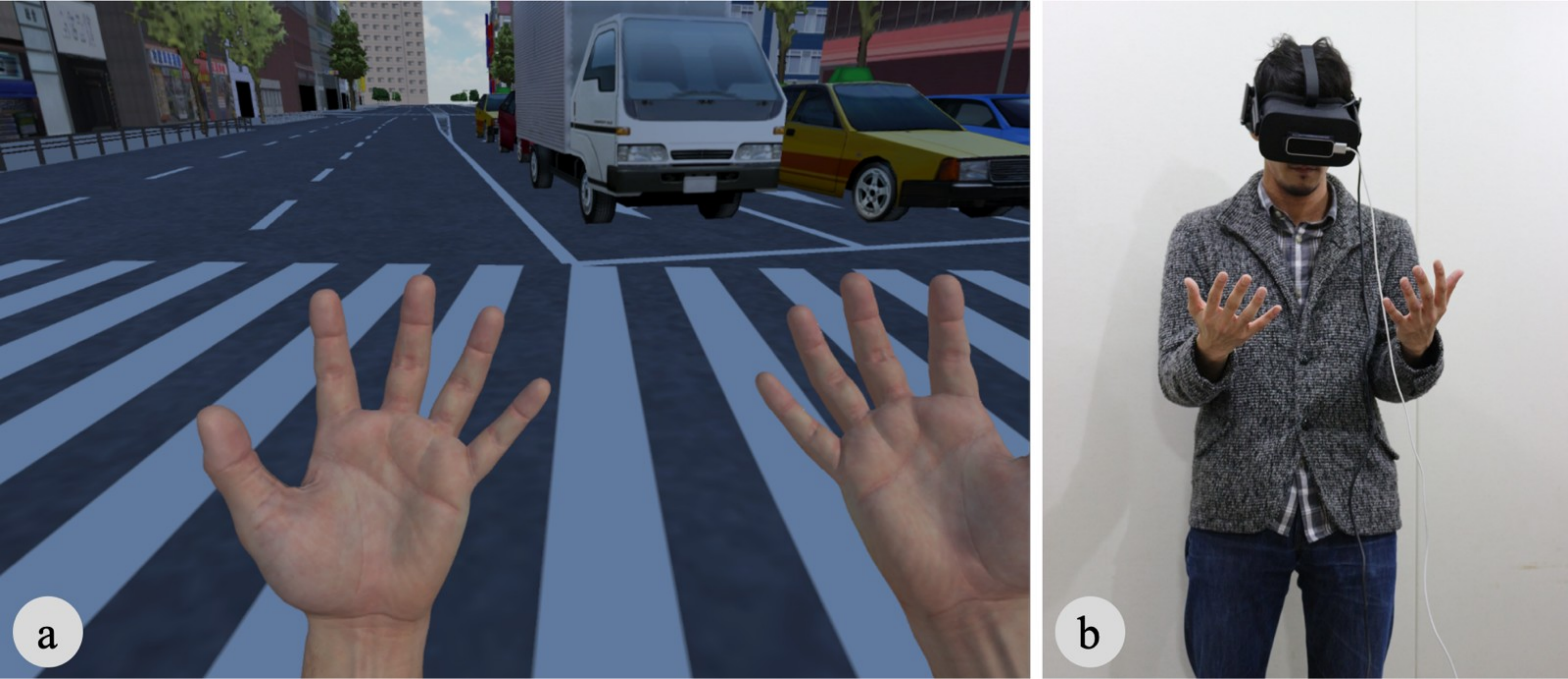
Experimental setup. The visual stimulus at the starting position was identical in all conditions (a). Participants were standing during the experiments and their hand movements were tracked by Leap Motion (b).

Participants were asked to stand in front of the experimenter, who fitted with them an HMD. They were instructed to remain standing and not to move from their initial position during the experiment unless they were given a break time. Then, when entering the virtual world, participants found themselves standing in the middle of an intersection in a virtual city (Fig 1). First, they were asked to move their hands freely in order to confirm that movement of the virtual hands was synchronized with movement of their real hands. They were also asked to report their perceived height of their body using a centimeter accuracy as an estimator of their perceived body size in the virtual world (e.g., 1.70m or 1.71m) (pre-Height). Next, the eye height was continuously increased up to twelve times higher than initial eye height (i.e., from 1.57m to 18.84m). Also, both the IPD and the hand size were manipulated in accordance with the increased eye height in each condition (see, Conditions in detail). During these manipulations, participants were instructed not to move their head and hands; they were told that their hands, as well as the truck on the road, must stay within the center of their visual field in order to control for fixation differences. When these manipulations were completed, participants were instructed to look around and move their hands freely. Then, they were asked to report their perceived height (post-Height) and their perceived eye height from the ground. The estimation of the perceived eye height was ten centimeters accuracy (e.g., 15.0m or 15.1m). The perceived eye height from the ground was assumed to represent the scale of the virtual world. Participants were asked to simply estimate the distance from their eyes to the ground, regardless of how they perceived their body size. The eye height, after the manipulations, was in the identical position relative to the virtual city model in all the conditions, hence the perceived eye height from the ground depended on the perceived scale of the world. Next, the experimenter asked participants to rate the statements in Table 1, and they answered verbally. After rating these statements, participants removed the HMD and took short break before starting a new trial.

**Table 1:** Statements of questionnaires (roughly translated from Japanese).

Number	Statement
S1 (Body size)	It feels as if my body has become larger.
S2 (Floating)	It feels as if my body is floating in the air.
S3 (City Shrinkage)	It feels as if the virtual city has become smaller.
S4 (Ownership)	It feels as if the virtual hands are part of my own body.
S5 (Self location)	It feels as if I am actually there in the virtual environment of the presentation.

S1-S3 were original items in our study. S4 was extracted from [13], and S5 from [14]. S5 was used only in Experiment 2.

**Fig 2:**
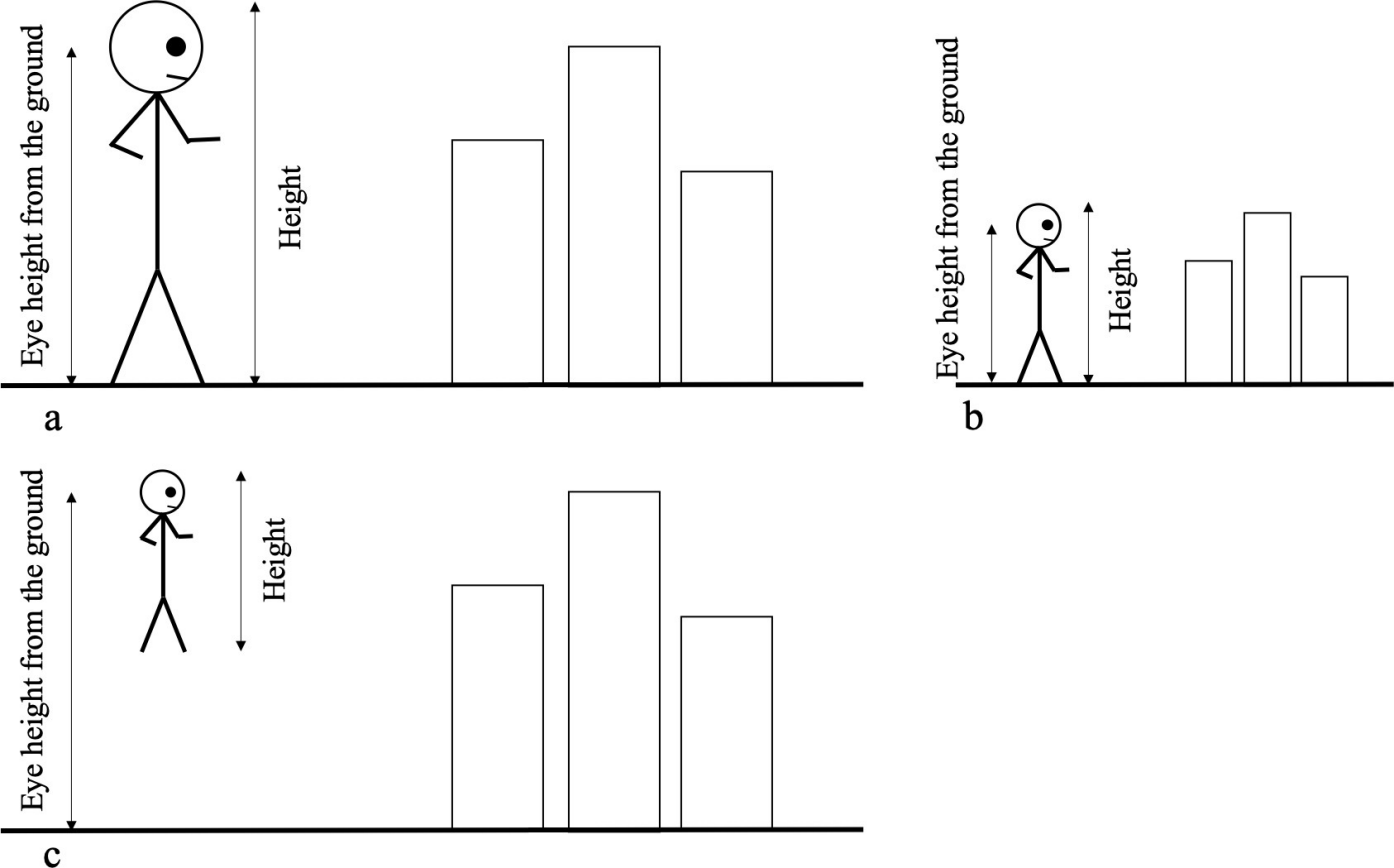
Relationship between the height and the eye height from the ground. Examples of the participants’ possible perceptions of their bodies and the external world. A giant in normal sized city (a), a normal sized person in a miniature city (b), a normal sized person in a normal sized city (c). In all conditions, the eye height after manipulations was in the identical position relative to the city.

We used the perceived height as an estimation of participants’ body size and the perceived eye height from the ground as an estimation of the scale of the city. Before manipulation, both of these indicators had to strongly correlate (i.e., perceived eye height must be almost 90% of the perceived height). However, when the eye height was vertically increased, these two indicators deviated according to participants’ perceptions of their bodies and the external world. If a participant perceives his or her body to be a giant (or normal sized) and the virtual city to be normal sized (or a miniature), then the indicators will not greatly differ (Fig 2A and 2B). However, if a participant perceives his or her body to be normal sized and the virtual city is also perceived as normal sized, then the perceived height will be the height of a normal sized person and the perceived eye height from the ground will be much higher than the perceived height as illustrated in Fig 2C.

#### Conditions

**Fig 3:**
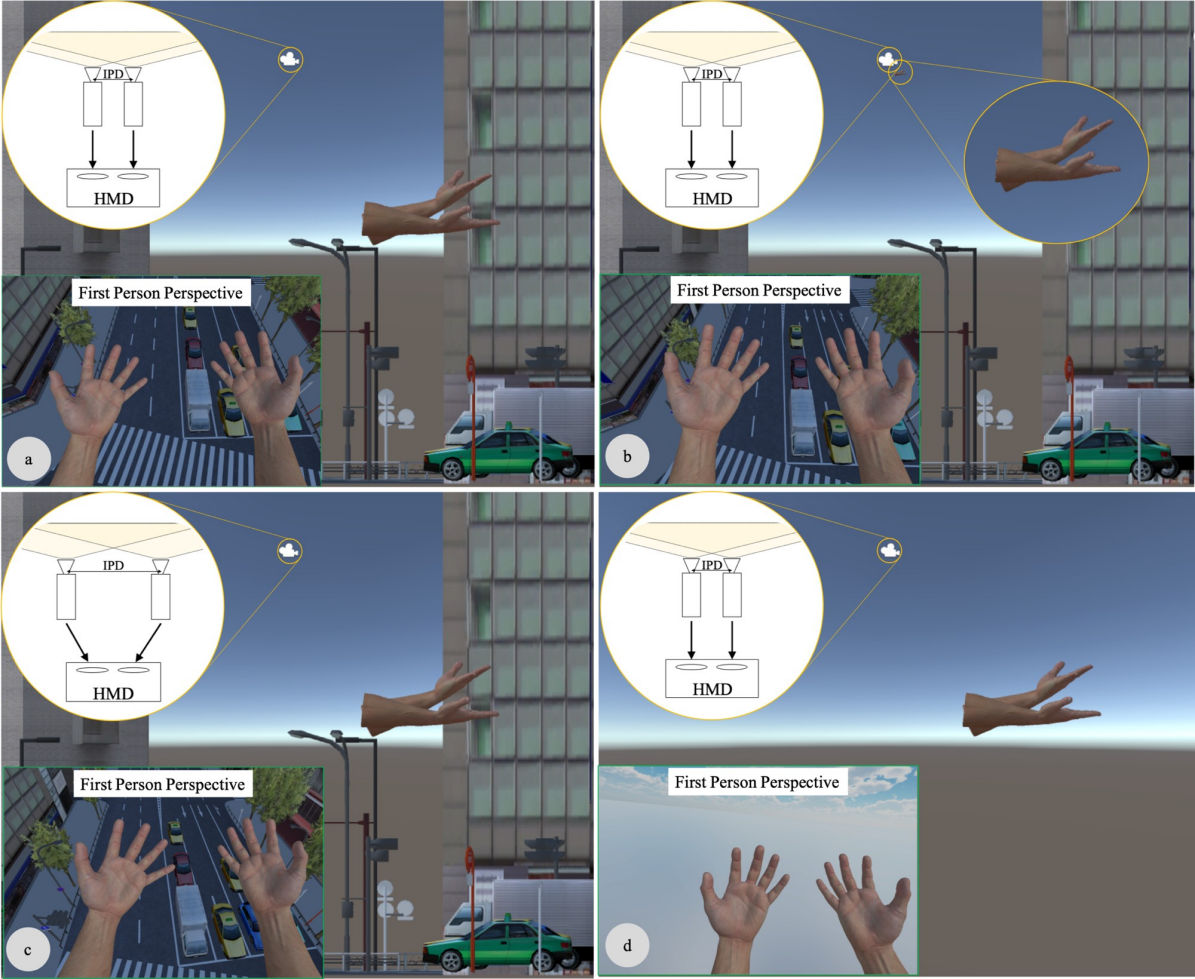
Experimental stimuli after manipulation. The Dynamic-hand condition (a), the Static-hand condition (b), the Dynamic-IPD condition (c) and the No-scene condition (d). In all but the Dynamic-IPD condition, participants’ IPD was fixed to 6.43 cm. The IPD was increased by up to twelve times in the Dynamic-IPD condition. The participants’ hands moved away from their eye position as their hands enlarged, so the size of hands on visual field is almost identical in all conditions.

This experiment comprised of four conditions. The Dynamic-hand condition in which we increased the participants’ eye height and their hand size while the IPD was fixed to 6.43 cm (Fig 3A). In this condition, a participant’s body size relative to the virtual city was increased up to twelve times larger than their normal size, although their IPD was fixed. The second condition, namely the Static-hand condition, involved a vertical increase of the participants’ eye height while their hand size and IPD were held constant (Fig 3B). These manipulations simulated that a normal sized person floating in the air (18.84m above the city). The third condition, the Dynamic-IPD condition, proportionally increased the participants’ eye height, hand size, and the IPD (Fig 3C) (These manipulations resembled the “Ground-Level Scaling” in Abtahi et al. (2019) [15]). In this condition, a participant’s body size, including the IPD to the scale of the virtual city, was increased up to twelve times larger than their normal size. The last condition is the No-scene condition in which we enlarged participants’ hands up to twelve times larger than their normal size, however, there were no visual cues except for their hands (Fig 3D). In all conditions, except for the No-scene condition, the eye height before the manipulation was the average standing height of 1.57m from the ground (In the No-scene condition, the ground was not presented, so the eye height could not be defined). Moreover, the participants’ hands moved away from the midpoint of their eyes as the hands enlarged (see, Fig 3), to induce the experience in which not only an individual’s hands, but his/her whole body, grew larger. By this manipulation, the visual angle with the hands was kept constant (see, Fig 3). All participants experienced all four conditions and the order of the conditions was randomized across the participants.

This experimental design aimed to compare three pairs of conditions. First, we compared the Dynamic-hand condition (Fig 3A) with the Static-hand condition (Fig 3B) to investigate how the size of body parts influenced the perception of the observer’s body and the external world when their IPD was fixed. Only hand size was manipulated between these two conditions. Kim & Interrante (2017) have shown that eye height does not affect the size perception of objects in the virtual world with rich visual cues [8]. Accordingly, in the present experiment, where the virtual city had plenty of visual cues (e.g. cars, buildings, traffic lights), participants should be predisposed to perceive little or no change in the scale of the virtual city between before and after this manipulation of visual information in the Static-hand condition. Moreover, because the size of their hands was static, individuals should not perceive their body size has changed; hence, individuals should perceive that they are floating above the ground in a normal-sized city (as if, for example, they were riding in a glass elevator) in the Static-hand condition. Therefore, we proposed to tell how participants perceive the scale of their body and that of the external world in the Dynamic-hand condition by comparing it with the Static-hand condition. If the body-based scaling robustly occurs in the Dynamic-hand condition, then the perceived scale of the external world should be smaller and perceived body size would be anticipated to change little, if any, compared to the Static-hand condition.

Next, we compared the Dynamic-hand condition (Fig 3A) with the Dynamic-IPD (Fig 3C) condition. The aim is to investigate how the IPD influences the perception of an observer’s body and the external world when a person’s hands are enlarged. In other words, only the IPD was manipulated between these two conditions. If one’s body is used as a metric that scales the external world, then it is assumed that there will be no difference in the size percepts of both the body and the external world experiences involving these two conditions. This is because the size of the body relative to the external world is identical in both cases. However, if the effect of the IPD on the perception of the external world [8–12] still remains in these conditions, then the perceived scale of the external world will be smaller in the Dynamic-IPD condition than in the Dynamic-hand condition. Consequently in this case the IPD will also affects their body size perception.

Following this, we compare the Dynamic-hand condition with the No-scene condition. The aim here is to examine how, in the absence of all other visual information except that of their hands, people perceive the scale of their bodies when their hands are enlarged. Only the visibility of the virtual city was manipulated between these two conditions. If people use visual cues in the external world to judge their body size, then they should not be able to perceive the change of the size of their hands in the No-hand condition.

#### Supplementary questionnaires

To further assess participants’ subjective feelings about their bodies and the external world, they were asked to verbally rate each of four statement items on a five-level Likert scale ranging from 1 (‘I don’t feel that at all’) to 5 (‘I feel that strongly’). Table 1 shows the statements we used in our experiments. We used statements S1-S4 in Experiment 1.

#### Statistical analysis

We defined the enlargement rate of a participant’s body as post-Height/pre-Height ratio. We compared three pairs of conditions involving, respectively, enlargement rate, perceived eye height from the ground, and the questionnaire data. However, the comparison between the Dynamic-hand condition and the No-scene condition concerned only the enlargement rate and the questionnaire data because perceived eye height could not be defined in the No-scene condition. All data were analyzed using the statistical software package R. In all comparisons using two conditions, we used Wilcoxon signed-rank test.

### Results

#### Dynamic-hand condition vs. Static-hand condition

**Fig 4:**
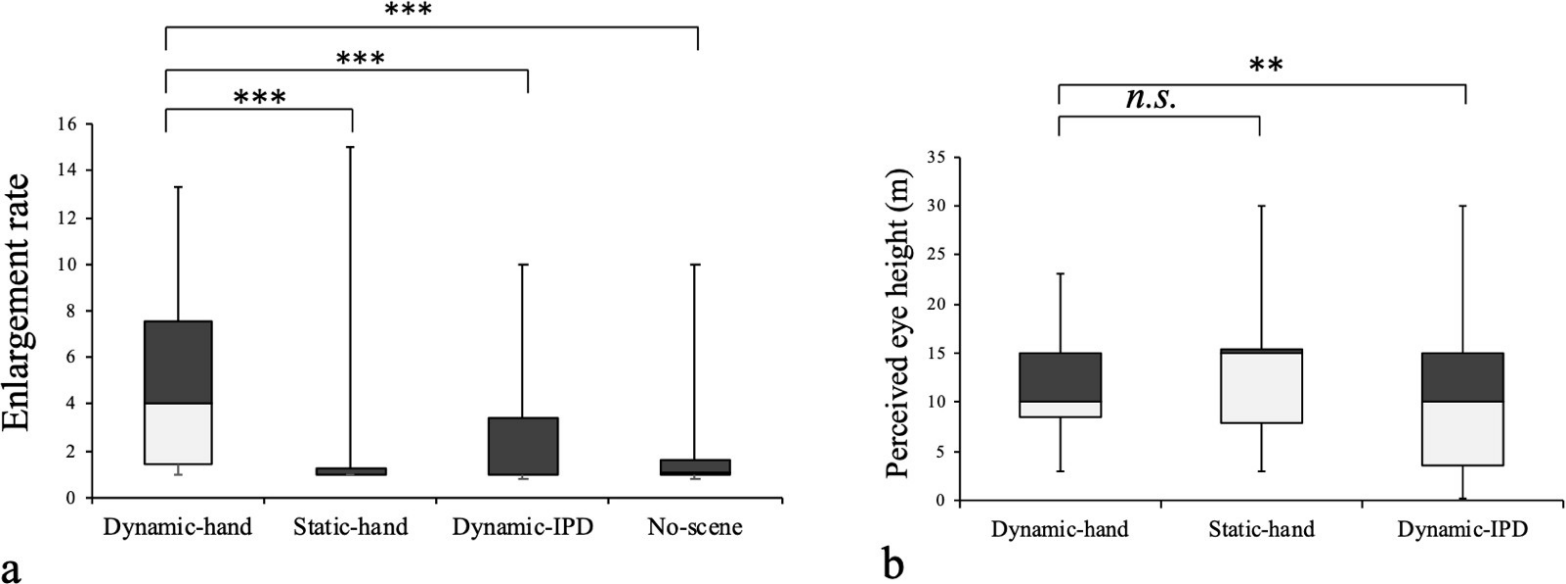
Results of Experiment 1. Two box plots of the enlargement rate of participants’ bodies (post-Height / pre-Height) (a) and the perceived eye height (b). The boxes are the interquartile ranges and the horizontal lines are the medians. The vertical lines indicate the maximum and minimum values. We compared the Dynamic-hand condition with the other conditions respectively. * p < .05, ** p < .01, *** p < .001 and *n*.*s*. p > .05.

First, we compared the Dynamic-hand condition with the Static-hand condition. The goal of this comparison was to investigate how people perceive their bodies as they experience their body parts becoming larger. The results showed that, when their virtual hands were enlarged, participants (in the Dynamic-hand condition) perceived that they were growing taller than in the Static-hand condition (*V* = 402, *p* < .001; see Fig 4A). Regarding the perceived eye height from the ground, there was no significant difference between these two conditions (*V* = 110, *p* = .60; see Fig 4B). The rating of S1 (Body size) was higher in the Dynamic-hand than in the Static-hand condition (*V* = 367, *p* < .001). The rating of S2 (Floating) was higher in the Static-hand than in the Dynamic-hand condition (*V* = 53.5, *p* = .0022). These results of the ratings of the statements are consistent with other results of estimates of participants’ involving height and the eye height from the ground. In the rating of S3 (City shrinkage) and S4 (Ownership), there is no significant difference between these two conditions (S3: *V* = 103, *p* = .070, S4: *V* = 39, *p* = .12).

As described above (see Conditions of Experiment 1), in the Static-hand condition participants should perceive the scale of the external world and their body size should not seem to change (i.e., they should merely seem to be floating above the ground). This response was supported by the very low ratings of S1 (Body size) and S3 (City shrinkage) in the Static-hand condition (median of S1 = 2, S3 = 1). Therefore, this implies that, in the Dynamic-hand condition, participants did not perceive the city to be smaller than in the Static-hand condition because a significant difference between these two conditions was not observed in the perceived eye height rating. These results suggest that participants in the Dynamic-hand condition perceived their body to be larger while they also perceived little or no scaling down of the external world. In conclusion, these results suggest that body-based scaling was not observed in the Dynamic-hand condition.

#### Dynamic-hand condition vs. Dynamic-IPD condition

Next, we compared the results associated with Dynamic-hand condition and the Dynamic-IPD condition. In this comparison, we investigated how one’s IPD affects an individual’s perception of body size. Previous research has shown that the IPD affects the scale perception of the external world, but it has not shown any influence of the IPD on body size perception. However, the present results showed that, in the Dynamic-hand condition, the enlargement rate was significantly higher than in the Dynamic-IPD condition (*V* = 394, *p* < .001; Fig 4A); also the perceived eye height was significantly higher in the Dynamic-hand condition than in the Dynamic-IPD condition (*V* = 307, *p* = .0034; Fig 4B). The rating of S1 (Body size) was higher in the Dynamic-hand than in the Dynamic-IPD condition (*V* = 241, *p* < .001). The rating of S3 (City shrinkage) was higher in the Dynamic-IPD than in the Dynamic-hand condition (*V* = 54.5, *p* = .0090). In the rating of S2 (Floating) and S4 (Ownership), there were no significant differences between these two conditions (S2: *V* = 193, *p* < .41, S4: *V* = 58.5, *p* = .14).

These results showed that participants perceived the external world to be smaller when their IPD was increased, a finding consistent with results of the previous research [8–12]. Moreover, these results suggest that the wider IPD led people to perceive less change in their body size than does static IPD, in spite of the fact that their hands became twelve times larger in both conditions. Thus, the IPD affects an observer’s body perception as well as the scale of the external world. These results suggest that our body is not always used as a fixed metric to scale the external world; rather, it is likely to be affected by the IPD.

#### Dynamic-hand condition vs. No-scene condition

Finally, we compared the Dynamic-hand condition and the No-scene condition in order to investigate the effect of the external environment on body size perception. The enlargement rate in the No-scene condition is significantly lower than in the Dynamic-hand condition (*V* = 518, *p* < .001; see Fig 4A). Also, in the No-scene condition, most participants did not perceive a change in their body size even though their hands grew to twelves times their normal size (the enlargement rate: Median = 1.01; see Fig 4A). The rating of the S1 (Body size) was higher in the Dynamic-IPD than in the No-scene condition (*V* = 351, *p* < .001). This result suggests that people cannot perceive a change in their body size when there are no visual cues in the external world. This corresponds to findings in prior research that suggest that size constancy does not work in a virtual environment filled with few visual cues [16, 17]. When there are insufficient visual cues, the distance between an object and the observer is difficult to judge. Thus, although the distance between participants’ eyes and their hands was increased in the No-scene condition, participants could not recognize this change. From these results, it seems that the information from the external world is crucial for people to judge their body size. In rating of the S4 (Ownership), there is no significant difference between these two conditions (*V* = 57, *p* = .38).

### Discussion

A major result of the present experiment reveals that people perceive a change of their perceived body size rather than the external world when their IPD is fixed. On the other hand, people perceived a change in the external world, instead of a change of their perceived body size, when their IPD is increased. From these results, it can be said that the body does not always function as a dominant scale, and the size of the body is strongly affected by the IPD. However, there is a possibility that the difference of the retinal size of participants’ hands created the observed difference of the perceived body size between the Dynamic-hand condition and the Dynamic-IPD condition. It is true that the retinal size of participants’ hands was slightly different between these two conditions, but we think this was not the cause of the observed difference. The manipulation was identical except for the visibility of the virtual city between the Dynamic-hand condition and the No-scene condition. If the slight difference of the retinal size of hands induces the experience that one’s body is a giant in the Dynamic-hand condition, then participants should also perceive their body to be large in the No-scene condition because the retinal size of the hands was identical in these two conditions. However, most of the participants did not perceive such a change in their body in the No-scene condition. Thus, the difference of the retinal size of participants’ hands induced by the difference of the IPD did not have an effect on the body size perception.

Participants’ percepts in the Dynamic-IPD condition were partly inconsistent with the results of Langbehn et al. (2016). These investigators found that both body and the environment can function as metrics that rescale each other. Furthermore, in their study no consistent tendency among participants was observed when participants’ eye height, their IPD and their hand size were proportionally increased (almost identical to the Dynamic-IPD condition) [7]. However, Langbehn and colleagues used a two-alternative forced choice task that forced participants to judge which was the dominant scale, their body or the environment. Thus, in their study, participants had to choose one answer even when they could not detect the dominant scale (e.g., when they perceived both their body and the external world to be changed). Our results of the Dynamic-IPD condition showed that some participants simultaneously perceive the external world to be small and their body to be large, in spite of the fact that the enlargement rate is significantly lower than in the Dynamic-hand condition. It seems that participants’ alternative choice of the dominant scale in [7] may not accurately reflect their perception of their body and the external world.

Our results in Experiment 1 showed that people could not perceive the change of their body size when the external world had few visual cues, whereas they were able to perceive their body to be large when the world was filled with rich cues. Thus, it can be said that the information in the external world plays important role in judging the body size. Moreover, our results also showed effects of the IPD on scale perception of the external world. From these results, we can infer that people do compare their body relative to the external world, specifically they perceive the size of their body depending on the perceived scale of the external world as this scale is affected by the IPD. Therefore, in Experiment 2 we pursue the impacts of the IPD on body size perception and the relationship between our body and the external world. In this study we investigated the effect of the IPD on body size perception when one’s body is invisible. In this case, an individual cannot explicitly compare his or her body with the external world. Moreover, in Experiment 2, we used a size judging task as an indicator of the scale of the external world, in addition to the perceived eye height. Participants judged their eye height from the distance between their eyes and the ground, or from the height of an object located horizontally along same sight line as their eye height. The former was an egocentric distance, whereas the latter was an exocentric distance. A prior study suggests that egocentric and exocentric distance depend on respectively different visual cues [18]. Thus, the perceived eye height may be affected by certain information that participants used as a spatial cue. For a more accurate estimation of participants’ scale perception, we used a size judging task which has been typically used as an indicator of the scale perception of the external world.

## Experiment. 2

### Method

#### Procedure

**Fig 5:**
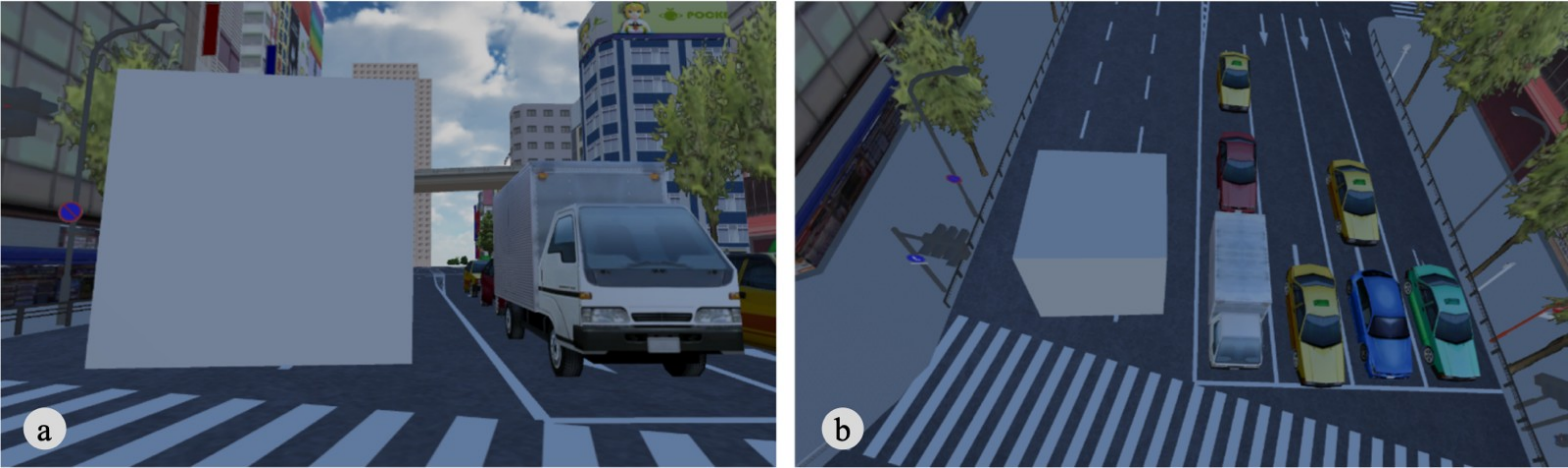
The cube in the virtual city. The view from the position before manipulation (a), and after manipulation (b). This figure illustrates the cube of 5m on each side.

The procedure of Experiment 2 was almost identical to that of Experiment 1 with the exception that participants in Experiment 2 estimated the size of cubes placed in the virtual world as an indicator of the perceived scale of the world, in addition to the perceived eye height. The cubes were placed next to a truck and located about five meters ahead from the participants’ starting position (Fig 5). Participants were asked to estimate the size of the cubes verbally using ten-centimeter accuracy (e.g., 5.0m or 5.1m) before the manipulation of the eye height (pre-size) and after the manipulation (post-size). We used two different sizes of cubes, i.e., 5m and 7m on each side, in order to prevent participants from recognizing that the size of the cubes was identical across conditions.

#### Conditions

The experimental design in Experiment 2 was a 2 × 2 factorial design (i.e., comprised of four conditions.) We employed two hand conditions (visible vs. invisible) crossed with two IPD conditions (normal vs. wide). The VH-NI (visible hand and normal IPD) condition was identical to the Dynamic-hand condition in Experiment 1. The VH-WI (visible hand and wide IPD) condition was identical to the Dynamic-IPD condition in Experiment.1. The IH-NI (invisible hand and normal IPD) condition was identical to the VH-NI condition except for hands which were invisible. The IH-WI (invisible hand and wide IPD) condition was almost similar to the VH-WI condition, but the participant’s hands were invisible. In all conditions, participants estimated both their body height, their eye height from the ground and the cube size. As described above, we used two different sizes of cubes. Thus, each of the four conditions comprised two trials; consequently, this experiment consisted of eight trials. The order of the trials was randomized across participants.

The main goal of Experiment 2 was to ascertain how the IPD and presence of body parts influence perception of body size. To this end, we manipulated participant’s IPD and the presence of their hands while vertically increasing their eye height. When the hands are visible, participants can estimate the ratio of the size between their body and objects in the external world. On the other hand, when their hands are invisible, participants cannot estimate this ratio because there are no visual cues about their body. If the perceived size of a viewer’s body is estimated by comparing it with the objects in the external world, then perceived body size will not be affected by the IPD. This is because the ratio of the size of the body to the external world is not clear, when the hands are invisible.

#### Supplementary questionnaires

To further assess the participants’ impressions, each participant was asked to verbally rate each statement on a five-level Likert scale ranging from 1 (I don’t feel that at all) to 5 (I feel that strongly). S4 was used only in visible hand conditions (VH-NI and VH-WI). Table 1 shows the statements used.

#### Statistical analysis

We defined the enlargement rate as post-Height/pre-Height and resizing rate of the cubes as post-size/pre-size. We took the average of each participant’s response (the enlargement rate, the perceived eye height from the ground, the resizing rate and the questionnaire data) over the two trials in each condition as the participant’s data of a given condition (e.g., a participant’s enlargement rate in the Dynamic-hand condition was the average of his/her enlargement rate of the two trials (5m cube and 7m cube) in the Dynamic-hand condition).

We employed the aligned rank transform (ART) procedure using the ARTool package in R [19] and conducted a two-way ANOVA for enlargement rate, eye height from the ground, resizing rate of the cubes and questionnaire data. Harrar, Ronchi & Salmaso (2019) suggested that ART performs well for testing both the main factor and the interaction of nonparametric data [20]. All data were analyzed using the statistical software package R.

### Results

#### Perceived body size

**Fig 6:**
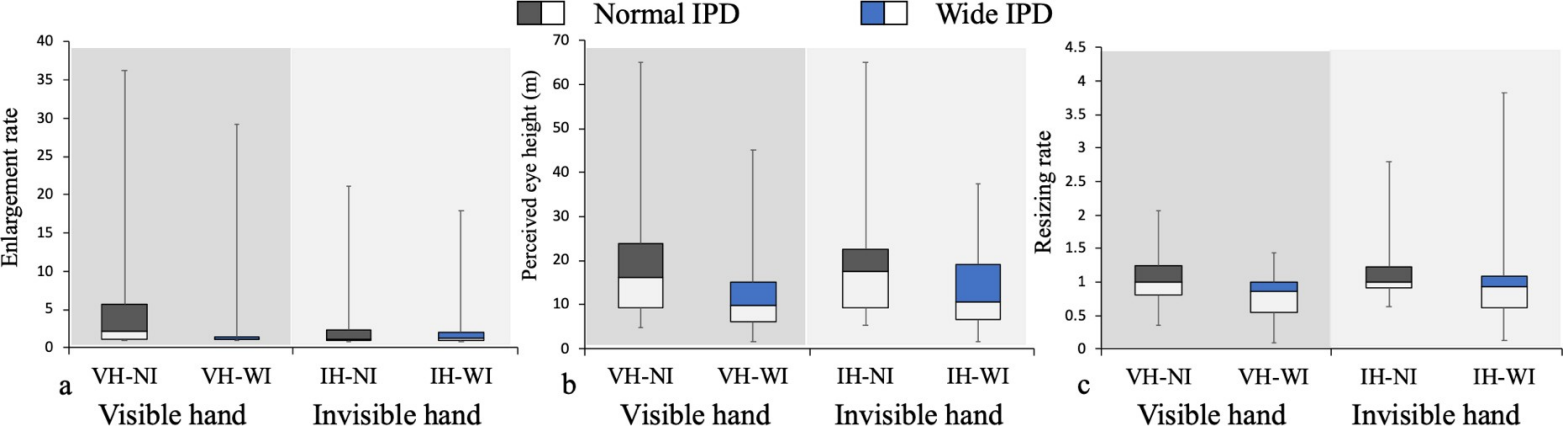
Results of Experiment 2. Box plot of the enlargement rate of participants’ body (post-Height / pre-Height) (a) and the perceived eye height (b) and the resizing rate of the cubes (post-size / pre-size) (c). The VH-NI condition is identical to the Dynamic-Hand condition, and the VH-WI condition to the Dynamic-IPD condition in Experiment 1 (see Fig 4). The boxes are the interquartile ranges and the middle horizontal lines are the medians. The vertical lines indicate the maximum and minimum values.

Regarding the enlargement rate (Fig 6A), results showed that a significant main effect of the IPD was observed (*F* (1, 23) = 5.70, *p* = .026). In addition, a significant interaction emerged between presence of hands and the IPD (*F* (1, 23) = 6.54, *p* = .018). A post-hoc analysis was conducted using the Bonferroni-corrected Wilcoxon signed-rank test for each pair of conditions. These analyses showed that, in the VH-WI condition, participants perceived their bodies to be smaller than in the VH-NI condition (*V* = 273, *p* < .001); this result is consistent with the result of the enlargement rate in Experiment 1. On the other hand, no significant difference emerged between the IH-NI and the IH-WI conditions (*V* = 64, *p* = .58). These results suggest that, when their IPD is increased, people perceive their bodies to be smaller than when their IPD is static, but this is true only when participants’ hands are visible. These analyses also showed that, in the IH-NI condition, participants perceived their body to be smaller than in the VH-NI condition (*V* = 223, *p* = .0083). No other significant effect was observed.

#### Perceived scale of the external world

Next we analyzed the data concerning the scaling of the external world. Both the perceived eye height and the resizing rate of the cubes were measured as indicators of the scale of the external world. Results showed that the main effect of the IPD was observed in the perceived eye height (*F* (1, 23) = 21.82, *p* < .001; see Fig 6B) and in the resizing rate of the cubes (*F* (1, 23) = 5.77, *p* = .025; see Fig 6C). These results are consistent with the result of the perceived eye height in Experiment 1. Both these indicators showed that, when the IPD is wider, people perceive the external world to be smaller irrespective of the presence of body parts. There was no other significant effect. Because we observed high similarity between the effect of the IPD on the perceived eye height and the resizing rate, it is fair to conclude that the perceived eye height may precisely indicates the scale of the external world as well as providing an estimate of the cube size.

#### Subjective impression

Finally, we analyzed the questionnaire data. In the rating of S1 (Body size), the results revealed a main effect of the presence of the hands was observed (*F* (1, 23) = 8.43, *p* = .008); also observed was a significant interaction between presence of hands and the IPD (*F* (1, 23) = 22.24, *p* < .001). We conducted Bonferroni-corrected Wilcoxon signed-rank test for each pair of conditions as post-hoc analyses. These analyses showed that, the rating of S1 (Body size) was significantly higher in the VH-NI than in the VH-WI condition (*V* = 197, *p* = .0029), and the rating was higher in the VH-NI than in the IH-NI condition (*V* = 166, *P* < .001). Concerning the rating of S2 (Floating), the results revealed a main effect of the IPD (*F* (1, 23) = 9.72, *p* = .0048), and an interaction between presence of hands and the IPD was also observed (*F* (1, 23) = 6.54, *p* = .018). We also conducted Bonferroni-corrected Wilcoxon signed-rank test for each pair of conditions as post-hoc analyses. These analyses showed that, the rating of S2 (Floating) was significantly higher in the IH-NI than in the IH-WI condition (*V* = 242, *p* < .001). With respect to the rating of S3 (City shrinkage), the results revealed a main effect of the presence of the hands was observed (*F* (1, 23) = 5.54, *p* = .028); also a main effect of the IPD was observed (*F* (1, 23) = 10.76, *p* = .0043). Results showed that participants subjectively felt that the external world became smaller when the IPD was wider and when their hands were visible. In the rating of S4 (Ownership), a significant difference was not observed between the VH-NI and the VH-WI condition (*V* = 52.5, *p* = .088). Also, the ratings of S5 (Self location) showed no significant effect.

### Discussion

We found that the IPD affects participants’ perception of body size only when the hands are presented. On the other hand, the IPD affects the scale of the external world regardless of the presence of hands. These results strongly support the hypothesis that people judge their body size by comparing it with the visual size cues in the external world. Thus, perceived body size is determined by a combination of perceived scales of the external world with the ratio of the size between one’s body and the external world when this ratio is clear. When body parts are invisible, people cannot use the information about relationship of the size between their body and the external world. Consequently, the difference of the perceived scale of the external world, induced by the difference of the IPD, does not affect the perceived body size. Although these results may have statistical limitations, due to small sample size, it should be noted that the effect of the IPD on the perceived height and the perceived scale of the external world that was observed in Experiment 1 has been replicated in Experiment 2.

## General discussion

The main result of our research is the finding that the perceived size of one’s body is affected by the perceived scale of the surrounding external, a scale that is strongly affected by the IPD only when perceiver’s hands are visible. Additionally, we found that our body is not always used as a metric to scale the external world. This discovery may be seen to conflict with the notion of body-based scaling, which claims that people use their own body as a metric to scale the size of the external world. However, in most research on this topic that appeals to body-based scaling, the perceived size of the body has not been a major focus. Consequently, we have not been able to determine how participants perceived their body size, given this research [3, 4] because we cannot easily compare these two situations. Yet, we believe these two constructs are not mutually exclusive; rather they are derived from the same mechanism. Both of them address the problem in that when the ratio between the size of one’s body and the environment changes, whether people may attribute this change to a change of their own body or to a change of the external world. Thus, when the conviction that one’s body size does not change is strong, then people will perceive that the external world changes, and vice versa. In a daily life, people cannot experience a sudden change of their body size, so the conviction that the size of one’s body does not change is likely to be strong. Thus, our bodies are used as a metric in many cases. However, if the belief that the scale of the external world does not change is also very strong, then their body can no longer used as the sole metric to scale the external world. Therefore, we may consider that the ways people judge how they attribute the change of the ratio involving their body and the external world to the change of their own body or to the change of the external world depends upon a combination of various factors that provide information about their own body and any external objects.

In our experiments, increased IPD appeared to considerably reduce individual’s belief that the external world does not change. Therefore, participants weighted their body, perceiving it as the dominant scale. On the other hand, most of the participants perceived the external world as a dominant scale in the Dynamic-hand condition in Experiment 1 (also in the VH-NI condition in Experiment 2). The manipulation that increases a participants’ eye height with the static IPD is not likely to induce an experience in which participants perceive the external world to be relatively small because this manipulation is identical to the situation in which people feel they are floating up above the ground. Moreover, using an outdoor scene as a virtual stimulus may contribute to promoting a belief that the external world does not change. There are, of course, various sizes of rooms in the world, so people do not find it strange to encounter a small room or large room. On the other hand, people probably have not experienced walking through a small scaled city in which cars, traffic lights, trees and other familiar objects are unusually small. For these reasons, participants will rarely perceive the change of the scale of the external world. However, some participants perceived the external world to be small and their body to be large at the same time in both the Dynamic-IPD condition (in Experiment 1) and the VH-WI condition (in Experiment 2). Related studies also suggest that people who are embodied in a large virtual body perceive the external world to be small; however, these participants also notice the change of their body size at the same time, although their estimation of their body size deviates strongly from the actual size of virtual bodies [5, 21]. These results suggest the possibility that neither one’s body nor the external world can provide an *absolute* metric to completely scale the other. If neither conviction, i.e., that the body does not change *and* the external world does not change, is not strong enough, then it becomes possible that we perceive that both of our bodies and the external world have changed at the same time.

Theoretically, in the Dynamic-hand condition and the Dynamic-IPD condition of Experiment 1(or the VH-NI condition and the VH-WI condition of Experiment 2), the enlargement rate was expected to be approximately “12” if participants perceive the scale of the external world not to change, and the perceived eye height should be approximately 1.57m if they perceive their body size as fixed. However, the median of the enlargement rate was “3.9” in the Dynamic-hand condition in Experiment 1 and “2.2” in the VH-NI condition in Experiment 2, although it is considered that the shrinkage of the external world hardly occur in these conditions. And the median of the perceived eye height was 10m and 9.8m in the Dynamic-IPD condition in Experiment 1 and the VH-WI condition in Experiment 2 respectively, although the median of the enlargement rate was “1” and “1.2”. One possible explanation for these unexpected outcomes appeals to prior knowledge. The familiar size of one’ own body and common objects placed in a virtual city. Investigations on the role of familiar objects indicate that they indeed affect both distance and size perception [22, 23]. Thus, participants’ perceptions of their body and of the external world were strongly influenced by the known size of these factors. If the external world featured no familiar objects, the perceived eye height can be expected to be lower than our results within the Dynamic-IPD condition of Experiment 1 and the VH-WI condition of Experiment 2.

Distance perception in virtual environments is slightly different from perception in the real environment; a number of studies address this disparity. Distance compression is a well-known phenomenon, that is, egocentric distances are underestimated in a virtual environment (see [24] as a review). In the present study, participants underestimate the distance from their eyes to the ground when their IPD was increased relative to when their IPD was fixed. We believe that this is because of convergence associated with the increased IPD. Moreover, in the present research although the eye height after manipulation was set to 18.54m, the median of participants’ estimation of the eye height from the ground was lower than 18.54m in all conditions including conditions in which their IPD was fixed. Therefore, distance compression was generally observed in our study. In other research, the impact of a manipulation of eye height on distance and size estimation in virtual environments has been suggested [25]. However, most of these studies featured environments with few visual cues. Kim & Interrante (2017) showed that the eye height does not affect the size perception of objects in a virtual world with rich visual cues [8]. In the present experiments, the virtual city had plenty of visual cues (e.g. cars, buildings, traffic lights), so it is assumed that the eye height had little or no effect on the scale perception of the external world. Moreover, even if there is an effect of eye height on scale perception, in our research this effect would have been controlled between all conditions because the manipulation of the eye height was completely identical under all conditions. Other studies have suggested that embodiment and accuracy of distance estimation have a positive correlation [26]. In the present study, the ratings of ownership (S4) which is among the major components of the embodiment were not significantly different between any pair of conditions. Therefore, accuracy of the distance estimation did not differ between conditions.

In our experiments, only the hands of a viewer were visible, not the full body. It is also possible that, in the conditions in which an individual’s hands were enlarged, these participants perceived that only their hands were larger while the size of their whole-body was not changed. However, in their feedback (after the experiments), none of the participants reported perceiving only enlargement of their hands. Furthermore, prior research has shown that abnormally transformed bodies considerably reduce ownership (e.g., an arm that is four times longer than normal [27]). Thus, if such viewers who perceive only their hands to be larger, the ownership on these hands would be reduced in those conditions in which their hands were enlarged. However, the rating of S4 (Ownership) did not significantly differ between the Static-hand condition, in which their hand size did not change, and the other conditions in Experiment 1. Thus, we conclude that participants perceived the size of their whole-body as proportional to their hand size, even though whole-body was not visible to the participants.

There are some limitations in the present research. First, we used only one scene as a stimulus. Therefore, there is a possibility that participants recognized that the virtual city was the same across conditions. Hence, the estimation of the scale of the external world might have been affected by the scale estimation in previous conditions and the differences of the scale of the external world between conditions would, therefore, be underrated. Second, we used an identical manipulation of the IPD and the eye height across participants. Ponto et al. (2013) investigated the effect of the calibration of viewing parameters (i.e., the distance between the rendering cameras and the position of the rendering cameras) on visual perception [28]. They showed that the calibrated parameters for accurate viewing are different across participants. Thus, visual perception of the manipulation of the IPD might differs across participants due to difference of the distance between pupils among participants. This point is also true for the eye height. The impression when experiencing the same manipulation of the eye height was different between participants because their physical eye height was different. The effect of the discrepancy between physical values and values of devices on the perception of the body and the external environment should be examined in future research. However, even if there were slight differences in the visual perception between participants in our study, this should not be of major concern because our study employed a within-participants design. Third, we focused only on a situation in which people are embodied in a large virtual body, so people’s perception of embodiment in a small body has yet to be investigated. Some studies imply that the impact of the IPD and eye height on size or distance perception is asymmetrical. For example, Kim & Interrante (2017) suggested that a smaller IPD may not lead people to perceive the external world as large, whereas a wider IPD can bias people to perceive the external world as small [8]. Leyrer et al. (2011) suggested that smaller eye height does not affect distance perception, although larger eye height affect distance perception [17]. This raises the possibility that people embodied in a small body may perceive their body or the external world differently than people embodied in a large body. This is a topic for future investigation. In any case, the paradigm we used in this research cannot resolve this issue. That is, a condition in which participants’ hands are shrinking while their IPD is static will not induce the body ownership to these hands because the hands are placed between eyes and this situation would be very awkward. Finally, in our experiment, participants did not interact with objects in the external world; they were allowed to move their hands freely, but no objects were placed within their reach. Some studies have implied that action capability or bio-energetic costs can affect the scale perception of the external world [4, 29–32]. Moreover, there are other non-visual cues that can affect the scale perception (e.g., appearance of an avatar [33]). Thus, the effect of these non-visual cues on body perception should be investigated in following studies.

It is interesting that participants perceived little change in the size of their own body in the Dynamic-IPD condition, even though these conditions simulated, most precisely, the experience of becoming a giant in the all conditions. On the other hand, most of the participants perceived their body to be large in the Dynamic-hand condition (the VH-NI condition). Therefore, the IPD should be fixed while the eye height is increased and the hands are enlarged when creating a virtual experience in which people could become much larger and taller.

Our research suggests that if one’s belief that the scale of the external world does not change is sufficiently strong, then the external world can be used as a metric to scale his/her body. This result will contribute the communication or interaction between multiple users who use various sizes of avatar with the same virtual environment. Gulliver and the Lilliputians saw the same world, but they perceived it from proportionally different size scales. However, if the scale of the world is different to each observer, then these observers cannot effectively use the units of distance. For example, a user perceives a distance from him/her to an object is 10 meters, but another user who is embodied in a differently scaled body perceives 100 meters. However, when the external world is used as a metric to scale our body, then the scale of the world is same between users, so they can effectively communicate and interact without contradiction in spatial perception of the world.

The present findings may be also applicable to clinical cases and the study of social behavior. Some research on gait rehabilitation has suggested that one’s walking pattern differs by using various gigantic avatars [34, 35]. Yee & Bailenson (2007) investigated that the effects of transformed self-representation on one’s social behavior [36]. They suggested that using taller avatars induce confidence in people. The effect of the changes of one’ body representation by manipulating the IPD on one’ behavior must be investigated in future studies.

## Conclusions

In the present study, we showed that the perceived size of one’s body is affected by the perceived scale of the surrounding external environment, a scale that is strongly affected by the IPD. This result means that one’s body is not always used as a metric to scale the external world, although many prior studies have suggested that the entire surrounding spatial layout rescales by changing body size. Although further research should be needed to clarify this point, one possible explanation is that both of them address the problem in that when the ratio between the size of one’s body and the surrounding external changes, whether people may attribute this change to a change of their own body or to a change of the external environment. Thus, when the conviction that one’s body size does not change is strong, then people will perceive that the external world changes, and vice versa. Finally, our finding that the perception of body size is affected by the IPD is novel and these results may contribute to the improvement of the interaction between multiple users in a virtual environment and to the clinical rehabilitations.

## Supporting information

S1 Fig Questionnaire results in Experiment 1. (TIFF)

S2 Fig Questionnaire results in Experiment 2. (TIFF)

S3 Fig Results of each participant for perception of their body in Experiment 1. (TIFF)

S4 Fig Results of each participant for perceived eye height in Experiment 1. (TIFF)

S5 Fig Results of each participant for perception of their body and the external world in Experiment 2. (TIFF)

S1 Data All relevant data to generate figures in our experiments. (XLSX)
