# Supplementary figures and images for: The relationship between the body and the environment in the virtual world: The interpupillary distance affects the body size perception
Source: PLoS One. 2020 Apr 24;15(4):e0232290. doi: 10.1371/journal.pone.0232290 (PMC7182220; doi:10.1371/journal.pone.0232290)

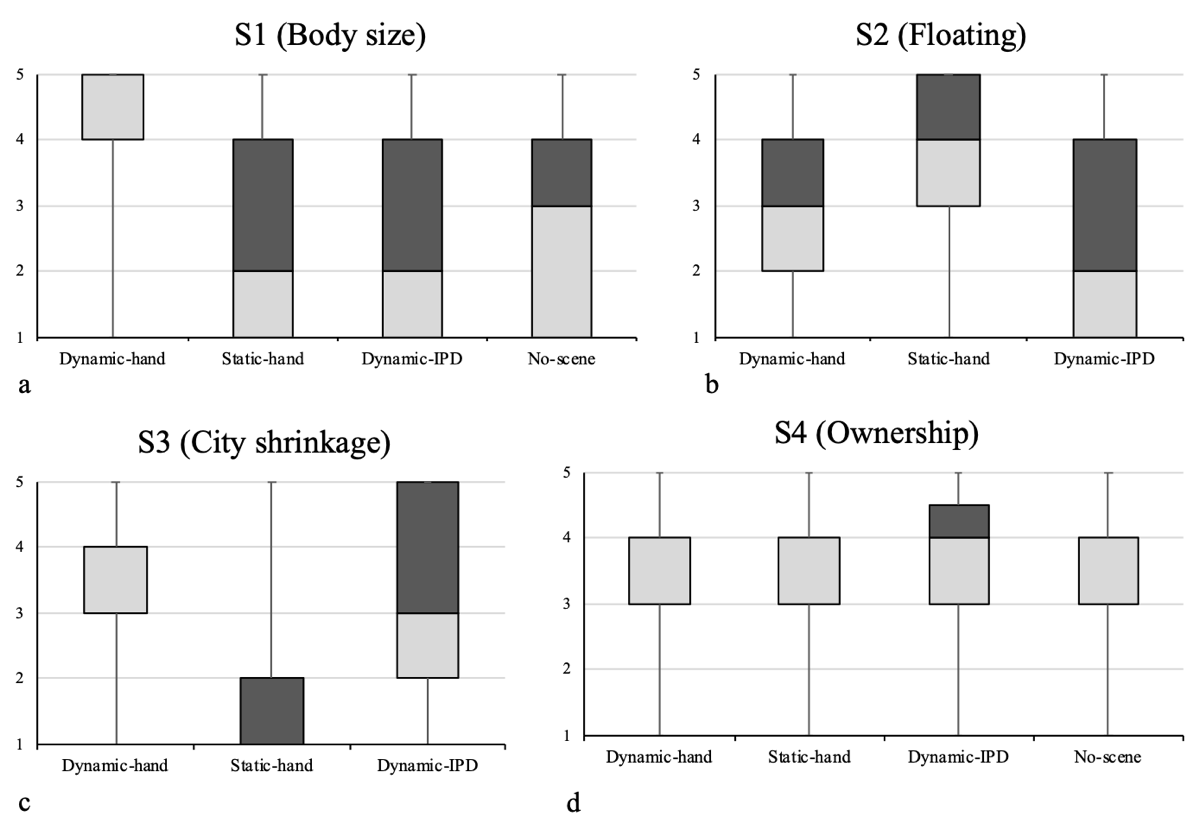

Supplement: S1 Fig — (TIFF) [file pone.0232290.s001.tiff]

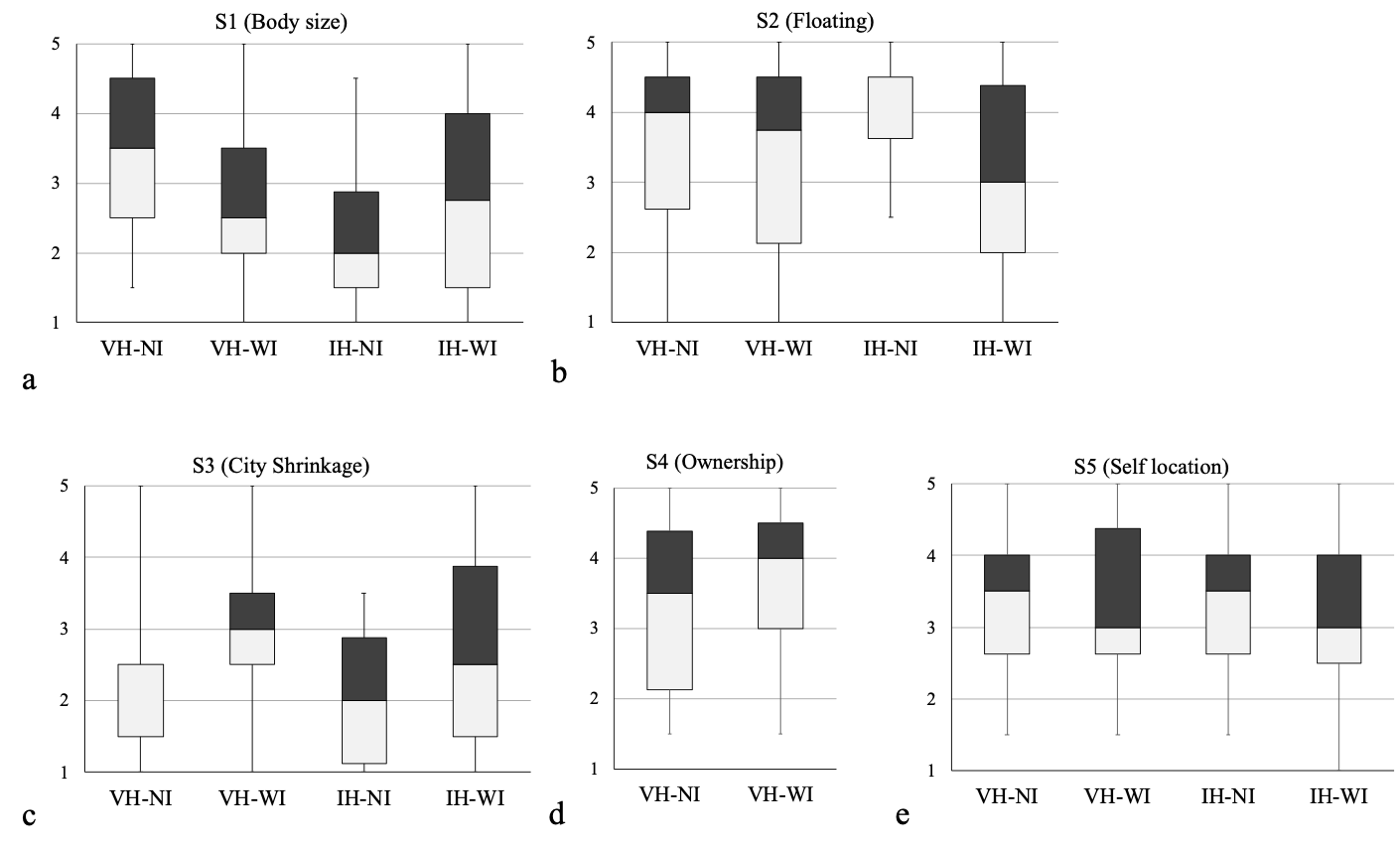

Supplement: S2 Fig — (TIFF) [file pone.0232290.s002.tiff]

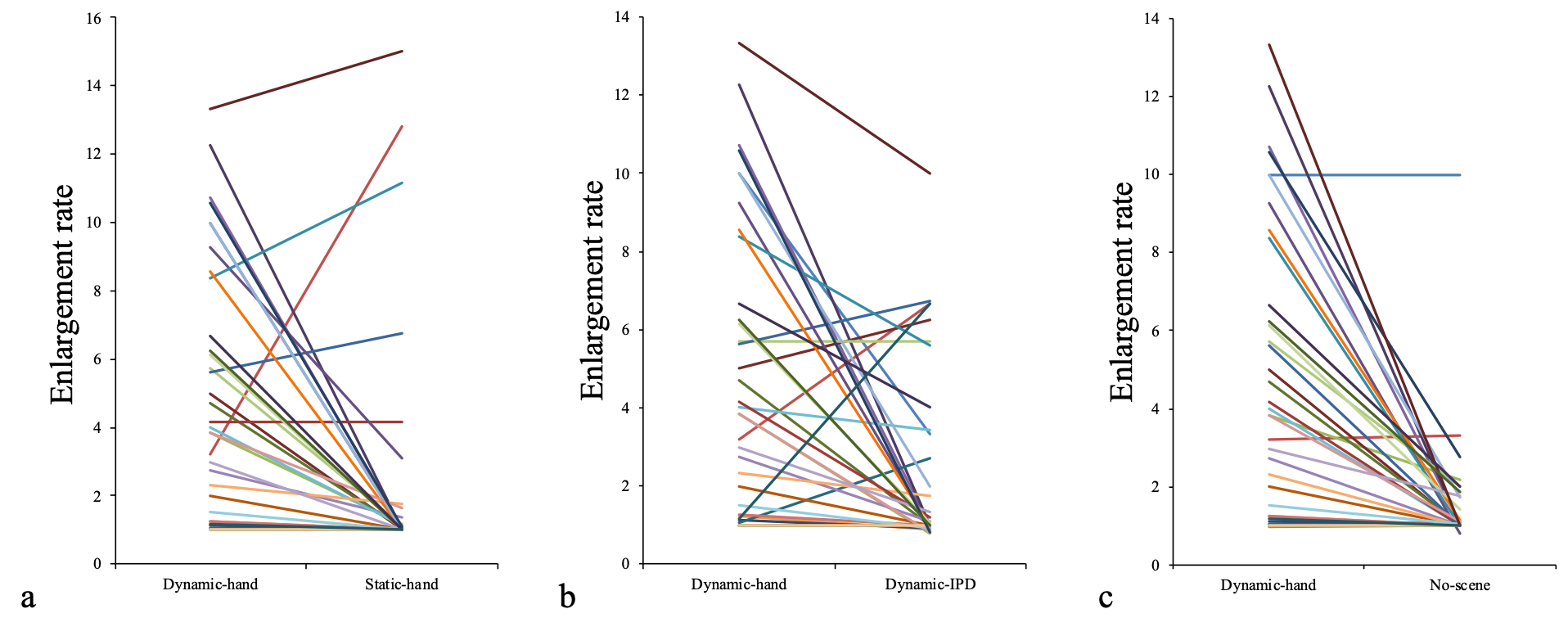

Supplement: S3 Fig — (TIFF) [file pone.0232290.s003.tiff]

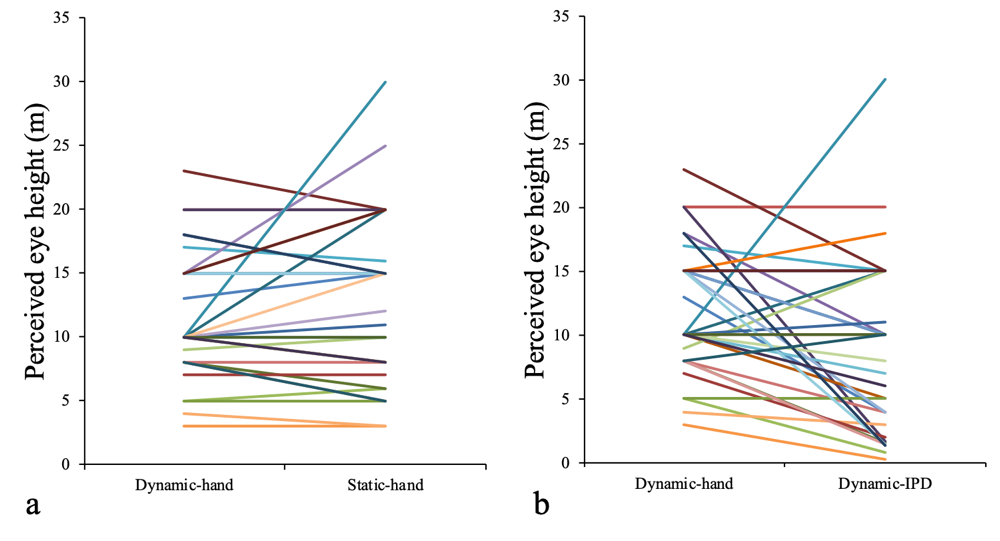

Supplement: S4 Fig — (TIFF) [file pone.0232290.s004.tiff]

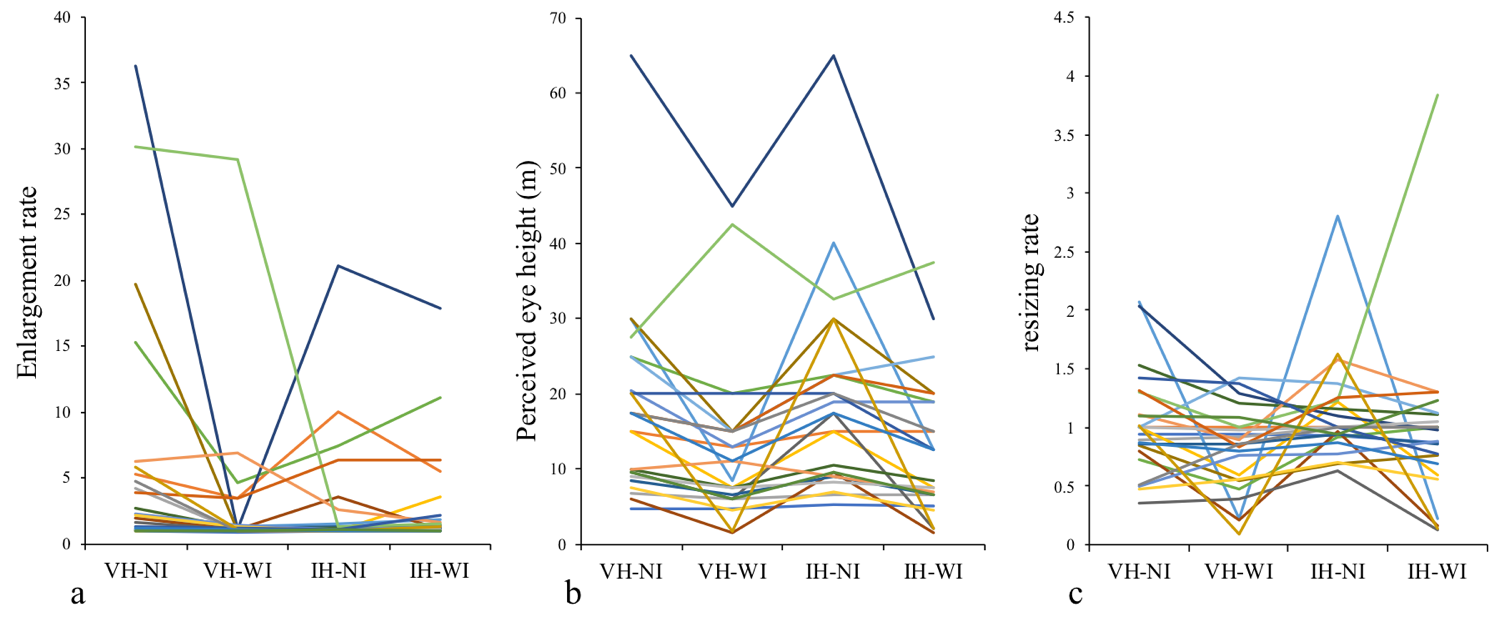

Supplement: S5 Fig — (TIFF) [file pone.0232290.s005.tiff]
